# Regulation of Cardiac-Specific Proteins Expression by Moderate-Intensity Aerobic Exercise Training in Mice With Myocardial Infarction Induced Heart Failure Using MS-Based Proteomics

**DOI:** 10.3389/fcvm.2021.732076

**Published:** 2021-10-08

**Authors:** Shouling Mi, Hao Jiang, Lei Zhang, Zhonglei Xie, Jingmin Zhou, Aijun Sun, Hong Jin, Junbo Ge

**Affiliations:** ^1^Department of Cardiology, Zhongshan Hospital, Fudan University, Shanghai, China; ^2^Shanghai Institute of Cardiovascular Diseases, Shanghai, China; ^3^Institutes of Biomedical Sciences, Fudan University, Shanghai, China; ^4^Stomatological Hospital, Fudan University, Shanghai, China

**Keywords:** aerobic exercise, mice, myocardial infarction, heart failure, proteomics

## Abstract

This study aims to systematically reveal the changes in protein levels induced by regular exercise in mice with ischemic-induced heart failure (HF). Aerobic exercise training for the ischemic-induced HF mice lasted for 4 weeks and then we used the liquid chromatography-mass spectrometry method to identify and quantify the protein profile in the myocardium of mice. As a whole, 1,304 proteins (597 proteins up-regulated; 707 proteins down-regulated) were differentially expressed between the exercise group and the sedentary group, including numerous proteins related to energy metabolism. The significant alteration of the component (E1 component subunit alpha and subunit beta) and the activity-regulating enzyme (pyruvate dehydrogenase kinase 2 and pyruvate dehydrogenase kinase 4) of pyruvate dehydrogenase complex and poly [ADP-ribose] polymerase 3, a nicotinamide adenine dinucleotide(+)-consuming enzymes, was further verified in targeted analysis. Generally, this proteomics profiling furnishes a systematic insight of the influence of aerobic exercise on HF.

**Graphical Abstract:**
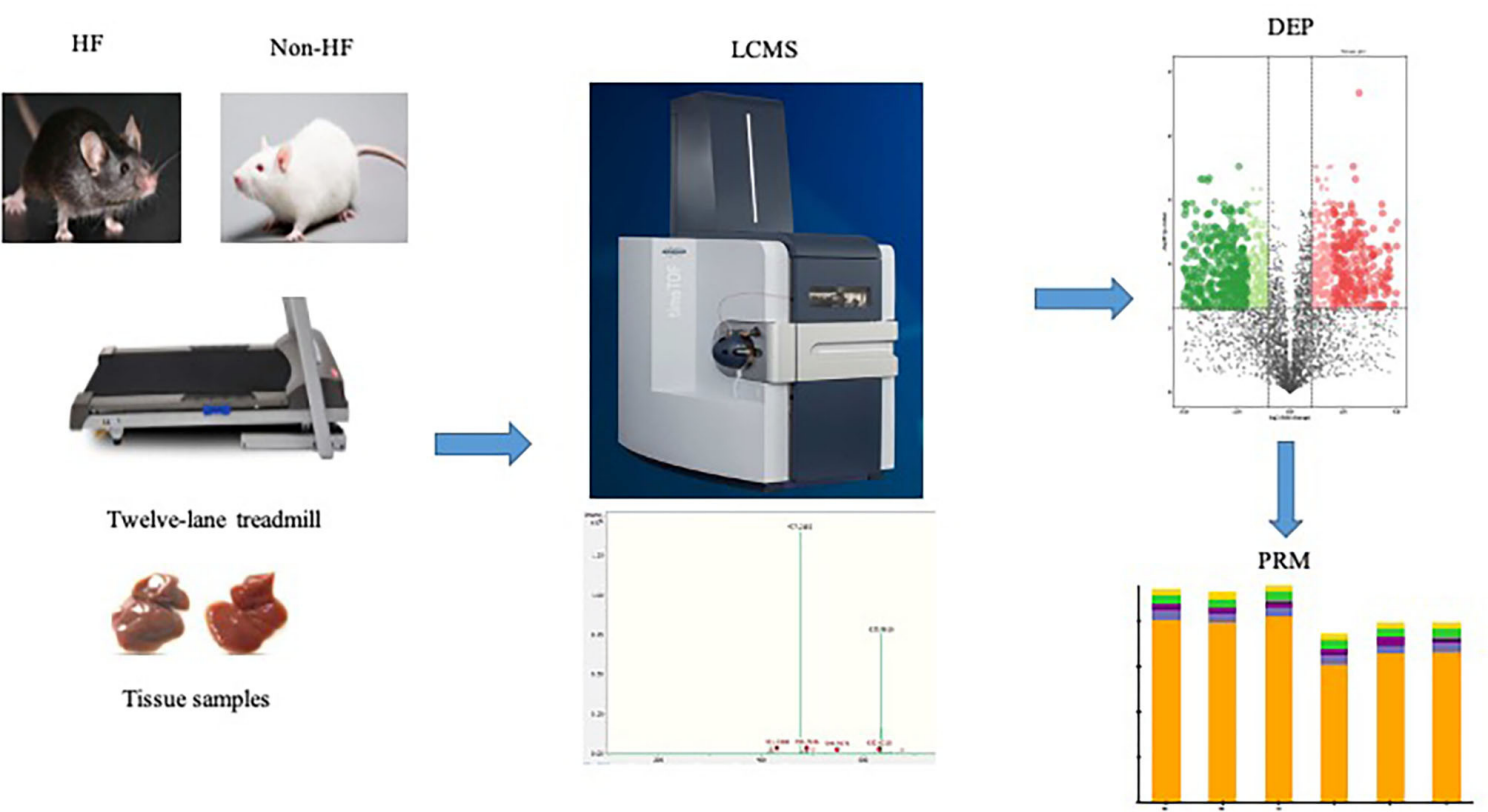
The heart failure model was induced by myocardial infarction in male C57BL/6N mice and then the mice with established HF were randomly assigned to the sedentary group or the exercise group in the 12-lane treadmill for 4 weeks. Myocardium samples of both exercise groups mice and sedentary groups mice were harvested, respectively. The LC-MS method was adopted for identification and quantification of the protein expression profile in the myocardium of mice. The retrieved protein peak area was used for subsequent statistical analysis. With the predetermined criterion (*P* < 0.05; fold change >2 or <0.5), 1,304 differentially expressed proteins were detected between the exercise group and the sedentary group, among which 597 proteins were up-regulated after exercise training and 707 proteins were down-regulated. Further targeted analysis was conducted to verify the expression changes in several key components of mitochondrial metabolism identified in the preliminary LC-MS analysis. HF, heart failure; LC-MS, liquid chromatography-mass spectrometry; DEP, differentially expressed protein; PRM, parallel reaction monitor.

## Introduction

With the increase of life-expectancy and underlying risk factors, heart failure (HF) is emerging as a major public health issue with the prevalence estimated to be 56 million patients worldwide (1). In the past few decades,pharmacotherapeutics has made great progress for HF from initial neuro-hormonal blockade of the cardiovascular system to additional neurohormonal regulation, as well as some recent anti-hyperglycemic drugs. However, the prognosis of HF patients remains less satisfactory. It is a challenge that the reductionist approach in this high clinical heterogeneity disease is due to the diverse therapy responding and treatment intolerance (2). Further multivariate therapeutic interventions are essential to improve the clinical outcomes of HF patients.

There is strong evidence that cardiac rehabilitation, especially the achievable non-pharmacological intervention training, could improve functional ability, relieve symptoms as well as reduce hospitalization, mortality, and ejection fraction for HF patients (3–7). Given the significant effectiveness and safety, training in the management of HF patients has been an alternative recommendation by authoritative guidelines (8, 9). Recently, more clinical trials began to focus on exercise intervention in different HF populations. For example, among HF patients with preserved ejection fraction, exercise training also yielded a beneficial effect on cardio-respiratory fitness and quality of life (10, 11) and the TOPCAT study further suggests that higher levels of physical activity have the potential to reduce the risk of adverse outcomes, which may attribute to the better indices of diastolic function (12, 13). In addition, exercise-based cardiac rehabilitation was also found to be feasible as an adjuvant treatment option with multiple HF groups with diverse complications (14, 15). That is to say, exercise may benefit more different subgroups of HF patients with discrepant pathophysiological settings, suggesting the multi-target effects of exercise training on HF condition. Experimental studies on the molecular mechanism of exercise intervention in HF may bring new enlightenment to the treatment strategies.

It was shown that several preclinical studies have already provided molecular insights for the beneficial effect of exercise in various induced HF animal models. As a whole, exercise training can ameliorate HF-induced dysfunctions by acting on the current standard pharmacological care-targeted pathways (16, 17) or non-pharmacological available targets correcting the inflammatory response, skeletal myopathy, and vagal outflow (16, 17). More recently, exercise was demonstrated to activate cardio-myogenesis in adult mice and the robust cardiomyogenic response was also observed in the adjacent area of the infarcted zone (18). Further, evidence indicates metabolic remodeling also contributes to the exercise-induced cardio-protection (19, 20). Chicco et al. demonstrated that low-intensity exercise could restore the mitochondrial energy metabolism via improving the activity of mitochondrial cytochrome oxidase (COx) and increasing the cardiolipin biosynthesis in the failing heart (20). In line with a previous result, it was also found that the cardiac function in myocardial infarction (MI)-induced HF model was improved after moderate-intensity exercise via upregulating mitochondrial respiration and glycolysis in our previous work (19). Just as the extensive effects in different HF groups, exercise training exerts its beneficial effects via different molecular mechanisms.

However, the abovementioned experimental trials were conducted in diverse animal models and the global alteration at the molecule level in an individual trial is scarce. Whether there is a critical target responsible for the effect of exercise on HF condition remains elusive. To date, no study has systematically revealed a global view for the alteration of protein expression regarding the cardiovascular adaptive response to exercise training in the setting of HF. Hence, in the present study, we used untargeted mass spectrometry (MS)-based proteomics to explore the moderate-intensity aerobic exercise-induced changes in expression level of proteins in mice with ischemic HF, aiming at a comprehensive understanding on the molecular basis for exercise-induced regulation of HF.

## Methods

### Experimental Animals and Models

In this study, only the male mice were used to avoid possible interference on exercise response from estrogen. Male C57BL/6N mice (21–25 g; 8–10 weeks) were purchased from Beijing Vital River Laboratory Animal Technology Co., Ltd. All experimental mice were treated in a uniform environment (22°C constant temperature; 12/12-h light/dark cycle; standard laboratory chow and tap water). The experimental procedures of animals strictly comply with the U.S. National Institutes of Health-published Guide for the Care and Use of Laboratory Animals (NIH publication no. 85–23, revised 1996). All experiments involving animals were reviewed and approved by the animal ethics committee at Zhongshan Hospital, Fudan University, China. Mice were subjected to either a surgery by ligation of the left anterior descending artery to induce MI and subsequent HF or sham surgery as previously described (21). All survived mice had echocardiography performed 1 week after surgery and mice with reduced left ventricular ejection fraction (EF <40%) were regarded as established HF. Then the mice with established HF were randomly assigned to sedentary group or exercise group. In addition, the same number of non-HF mice were also randomly divided into exercise groups and sedentary groups as controls.

### Aerobic Exercise Training and Materials Preparation

The 12-lane treadmill was utilized to conduct aerobic exercise 1 week after MI surgery. The first 3 days were the adaptative process of the exercise training in treadmill for mice (10 m/min; 1 h/day). After the adaptative process, regular exercise began at the fourth day and last 4 weeks (15 m/min; 1 h/day; 5 days/week). Mice that did not run in the treadmill were gently pushed and those manifesting the exhaustion condition (i.e., mice did not follow the running protocol even after 10 times gently push) were taken away from the treadmill to rest. The exercise capacity of all mice was tested by increasing the treadmill speed with a gradient of 5 m/min until exhaustion condition at the last training and the total running distance until exhaustion was recorded. Cardiac function of all mice was further tested by echocardiography after the exercise training and test process. Then, all mice were euthanized, and organs were dissected and rapidly frozen in liquid nitrogen.

### Sample Preparation for Mass Spectrometry-Based Quantitative Proteomic Analysis

The samples were combined, shredded in phosphate buffer saline, and ground with liquid nitrogen to mix. The lysate (7 M urea, 2 M thiourea, 0.1% phenylmethanesulfonyl fluoride-a protease inhibitor, 65 mM dithiothreitol [DTT]) was added at a ratio of 5:1 (lysate volume: sample weight) and then suspended and cracked on ice for half an hour. Next, samples were centrifuged at 12,000 g for 15 min. The supernatant was extracted, and the protein content was determined by the Bradford method. Two hundred microgram protein dissolved in lysate was taken to react with 10 mM DTT at 37°C for 1 h, 20 mM iodoacetamide in the dark at room temperature for 0.5 h and 4 times the volume of ice acetone for overnight precipitation at −80°C. The supernatant was extracted after centrifugation at 12,000 g for 15 min and resuspended with the 200 UL 50 mM ammonium bicarbonate. Trypsin was added at 1:50 for enzymolysis overnight at 37°C, which was terminated when the final concentration of formic acid was added to 5%. Finally, the final samples were desalted with the Sepak desalination column (Waters, U.S.) and prepared for mass spectrometry analysis.

### Inverse Separation at High pH

A 100-ug protein was extracted for high pH 2D separation on chromatographic column (BEH C18,300 Å, 1.7 um, 2.1 mm × 150 mm; Waters, U.S.) and concatenated into 20 fractions, which were finally combined into 4 fractions. Mobile phase A is water with 5 mM ammonium formate (pH 10.3), and B phase is acetonitrile. Within 20 min, B phase increased linearly from 5 to 45%. The fractions were lyophilized and redissolved in an aqueous solution of ‰ formic acid.

### The Chromatographic Conditions

We performed the second-dimensional separation using nano Elute liquid chromatography (LC) system (Bruker Daltonics). A 250 mm × 75 um column (Inopticks) was employed, in which the mobile phase A and B were water and acetonitrile of ‰ formic acid, respectively. Peptide separation was conducted at a flow rate of 300 nL/min within 90 min. The mobile phase B concentration increased from 2 to 22% in the first 45 min, followed by an increase to 37% within 5 min and another increase to 80% within 5 min before the last 5 min for maintenance rinsing. A 200-ng peptide fragment was used for LC-MS analysis.

### Mass Spectrometry Conditions

All fractions were analyzed by a hybrid trapped ion mobility spectrometry (TIMS) quadrupole time-of-flight mass spectrometer (TIMS-TOF Pro, Bruker Daltonics) using a nano electrospray ion source with a scanning range of 100–1,700 m/z and a trip range of 0.7–1.3 VS/cm^2^. The collection time of a single cycle was 1.16 s comprising 1 MS scan and 10 PASEF secondary scans. The intense threshold is 5,000 and we set the accumulation and release time as 100 ms, ion source voltage as 1,500 V, the auxiliary gas as 3 L/min, and the temperature of the ion source as 180°C.

### Data Analysis

The data were analyzed by Peaks Online software (Bioinformatics Solutions, Inc.) with the mouse database downloaded from SwissProt (17,046 proteins, 20,200,820 download). The MS1 error was 15 ppm, the MS2 error was 0.05 Dalton, and the trypsinase was set at half enzyme digestion. Carbamido-methylation protein C-term was set as fixed modification and acetylation (protein N-term), oxidation (M) and deamidation (NQ) were set as variable modification. The retrieved protein peak area was used for subsequent statistical analysis.

### Statistical Analysis

Proteins with an overall missing value of more than 50% are removed, and the remaining blank values are filled with a random number between 0 and the minimum area. *T*-test and fold-change values of proteins were used to screen differential proteins, and the GO (gene ontology) was analyzed using R package clusterProfiler, version 3.16.1.

### Verify the Differentially Expressed Proteins

To verify the up and down regulated proteins, we use the prmPASEF method based on the timsTOF Pro mass spectrum (22). For each protein, we choose a unique and high scored peptide to perform the prmPASE experiment; then check the peptide areas manually, and the areas are exported to plot the histogram.

## Results

### Study Overview

Aerobic exercise training for the ischemic-induced HF mice lasted for 4 weeks, and then myocardial samples of both the exercise group and sedentary group were harvested, respectively. Significant improvement in cardiac function was observed in the HF-exercise group compared with the HF-sedentary group as has been demonstrated in our preliminary work, including EF, fractional shortening, and exercise endurance (19). All samples were pre-separated by high pH reverse phase chromatography after enzymolysis, and each group of samples was finally combined into four fractions. The LC-MS method was adopted for further identification and quantification of the protein expression profile in the myocardium of mice. As a result, a total of 6,297 proteins were steadily identified in this experiment, among which proteins with missing values greater than 50% were directly deleted and the remaining blank values were filled by KNN algorithm, finally retaining 4,260 proteins for further differential analysis (Supplementary Table 1).

### Evaluation of the Proteomic Results

**Figure 1:**
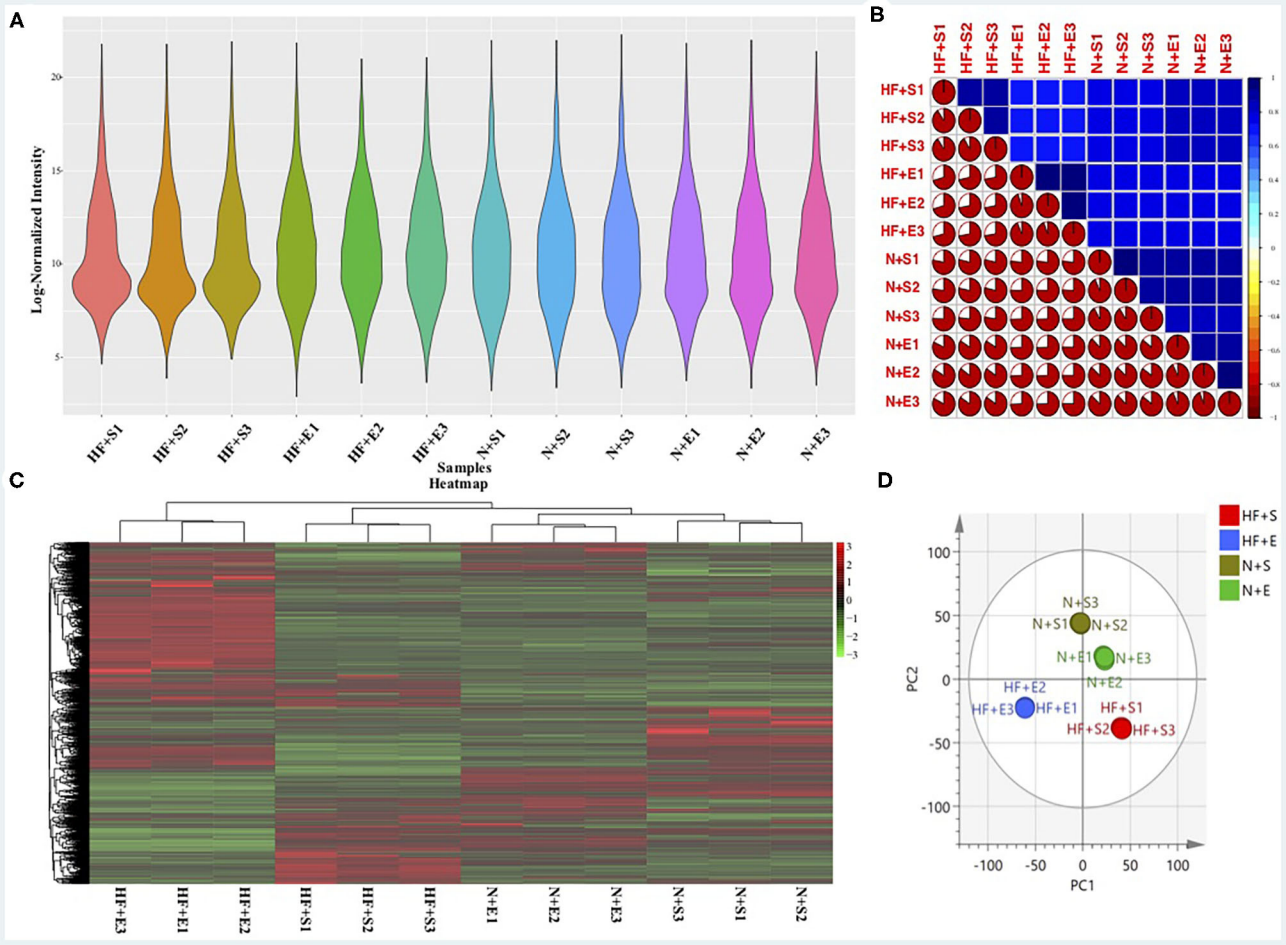
The sensitivity and reliability of the LC-MS results. **(A)** The peak intensity distribution of all samples. **(B)** The correlation of all samples using the peak intensity data. **(C)** Heatmap of differential expressed proteins within the four experimental groups and hierarchical clustering analysis. **(D)** O2PLS-DA of the four experimental groups by the identified protein data. LC-MS, liquid chromatography-mass spectrometry; HF+S, sedentary groups of heart failure mice; HF+E, exercise groups of heart failure mice; N+S, sedentary groups of non-heart failure mice; N+E, exercise groups of non-heart failure mice.

Reliability of the LC-MS results was evaluated. The peak intensity distribution of samples was exhibited in Figure 1A. We observed that the intensity distribution of 4,260 identified proteins spanning 6 magnitude orders,which was almost similar and balanced among these different groups, indicating the high reproducibility and sensitivity of the LC-MS method. Moreover, reproducibility of the results was also evaluated by calculating the correlation of peak intensity between any two samples among exercise groups or sedentary groups. As presented in Figure 1B, the adopted LC-MS method revealed excellent repeatability and reliability in the identification and quantification of protein expression profile in different samples with an average correlation coefficient (R package corrplot, version 0.84) of 0.8. The exercise group and sedentary group could be exactly divided according to the hierarchical clustering analysis (Figure 1C) and principal component analysis (Figure 1D) based on the quantitative data of identified proteins.

### DEPs and Pathway Analysis

**Figure 2:**
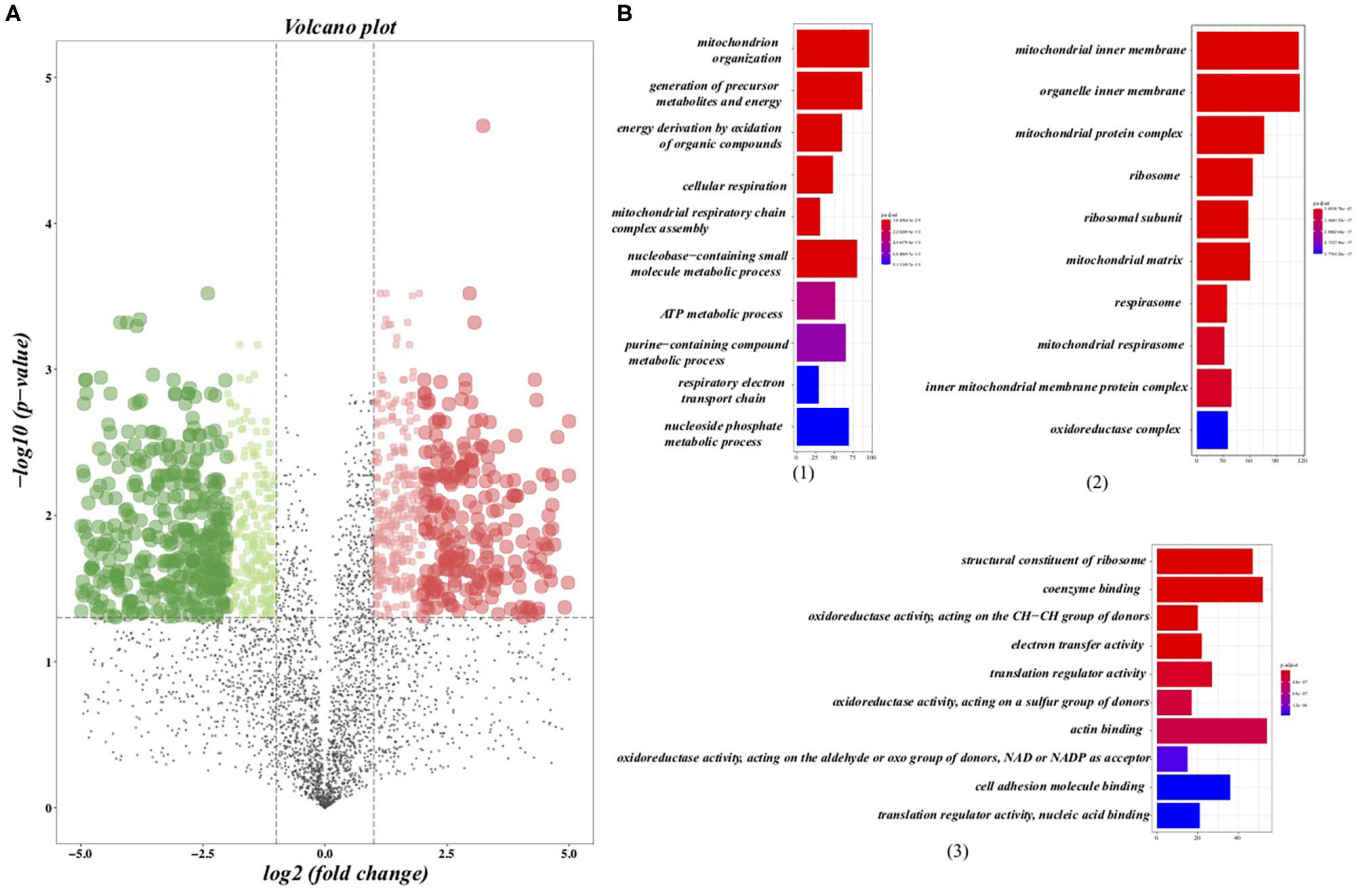
Differentially expressed proteins (DEPs) and the gene ontology (GO) analysis. **(A)** Volcano plots presented the DEPs between the sedentary group and the exercise group in HF mice, in which the x axis presented the fold change and the *y*-axis presented the *p*-value after adjustment. The dotted lines show selection threshold (adj. *P* < 0.05; fold change >2 or <0.5). **(B)** GO analysis: (1) Top 10 of enriched biological processes of DEPs; (2) Top 10 of enriched cellular components of DEPs; (3) Top 10 of enriched molecular function of DEPs.

We further analyzed the alterations of the protein expression profile induced by regularly aerobic exercise in ischemic-induced HF mice following the predetermined criterion (adj. *P* < 0.05; fold change > 2 or < 0.5) (23). As a whole, 1,304 proteins were differentially expressed between the exercise group and the sedentary group (Figure 2A), among which 597 proteins were up-regulated after exercise training and 707 proteins were down-regulated (Supplementary Table 2). GO analysis (Figure 2B) for the biological process revealed that these DEPs play crucial roles in material and energy metabolism especially the mitochondrial-related energy metabolism. Indeed, the cellular components analysis (Figure 2B) for the altered proteins further verified that those DEPs were significantly enriched in numerous structural and functional components of mitochondria, including the matrix and inner membrane of mitochondrial, mitochondrial respirasome, mitochondrial protein complex, and inner mitochondrial membrane protein complex. Moreover, ribosome and ribosomal subunit, the molecular machinery for protein synthesis in cells, are also significantly enriched by differential proteins. Whereas, the molecular function analysis (Figure 2B) indicated that those DEPs were associated with oxidoreductase activity (acting on the aldehyde or oxo group of donors, NAD or NADP as acceptor), electron transfer activity, and translation regulator activity. In summary, our research provided a comprehensive elucidation for the impact of regular exercise on multiple proteins in the ischemic-induced HF mice model. Metabolic-related pathways especially the energy metabolism pathways were significantly regulated by aerobic exercise training in the HF condition, which usually was characterized by insufficient energy supply.

### Verification of the Expression Changes of Key Proteins Involved in Energy Metabolism

**Figure 3:**
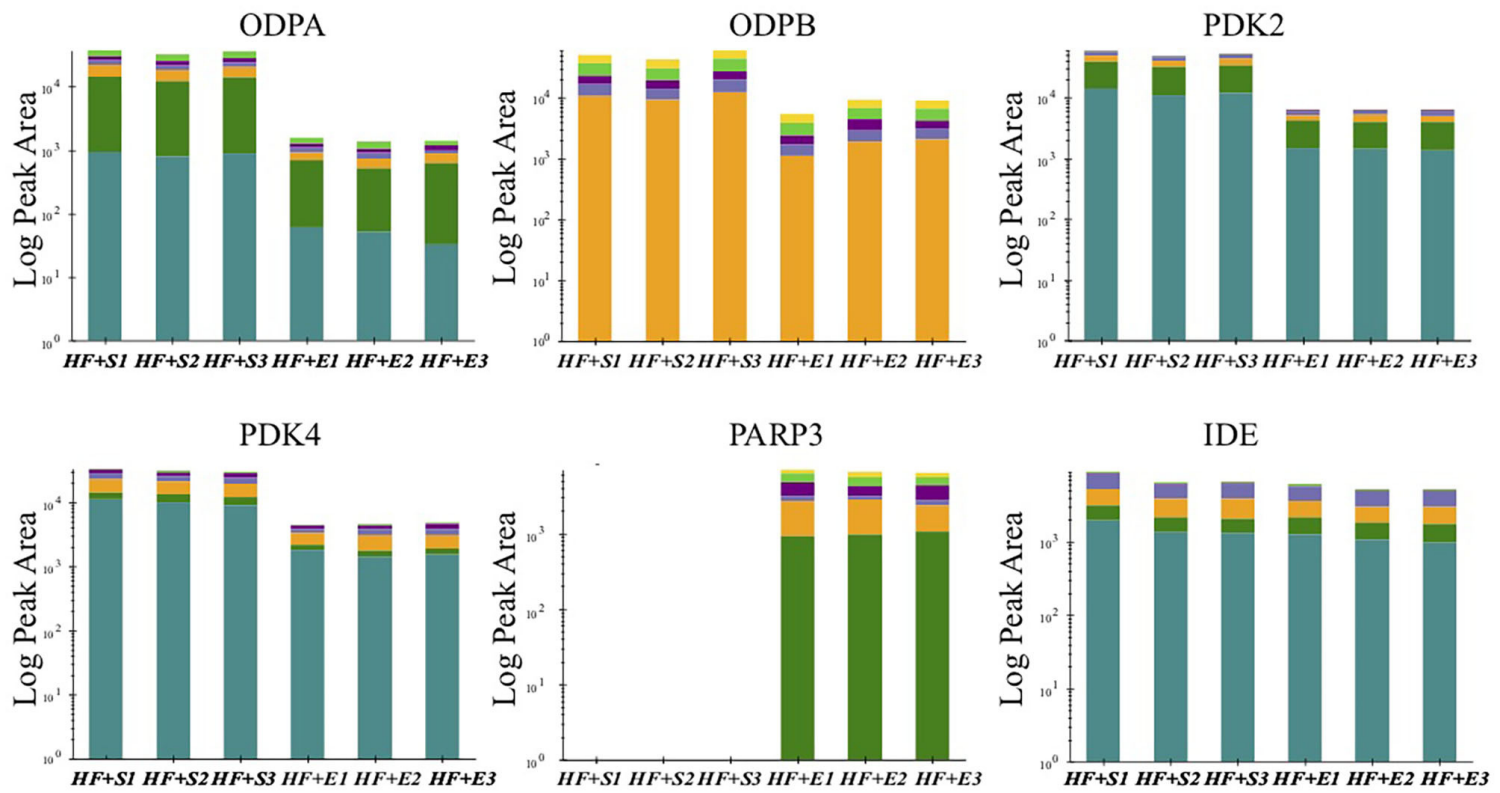
The peak area of verified proteins involved in energy metabolism in the sedentary group and the exercise group of HF mice. ODPA, pyruvate dehydrogenase E1 component subunit alpha; ODPB, pyruvate dehydrogenase E1 component subunit beta; PDK2, pyruvate dehydrogenase (acetyl-transferring)] kinase isozyme 2; PDK4, pyruvate dehydrogenase (acetyl-transferring)] kinase isozyme 4; PARP3, poly [ADP-ribose] polymerase 3; IDE, insulin-degrading enzyme.

The previous analysis has found that a variety of altered proteins is related to mitochondrial metabolism, which is usually impaired in a state of HF. Further study of the key molecules in this vital transition may provide more intuitive mechanism insight into the metabolic remodeling process of HF induced by aerobic exercise. Therefore, we conducted targeted MS analysis to verify the expression changes in several key components of mitochondrial metabolism identified in the preliminary LC-MS analysis. Finally, we observed that the expression level off our proteins involving the metabolism of adenosine triphosphate (ATP) and nicotinamide adenine dinucleotide (NAD) had consistent direction with the discovery stage (Figure 3). The expression of pyruvate dehydrogenase complex E1 component subunit alpha (PDC E1α) and subunit beta (PDC E1β) were both downregulated to almost the normal level especially the PDC E1β in the exercise-treated HF mice compared with the sedentary groups. Two phosphorylase isoforms of PDC, the pyruvate dehydrogenase kinase 2 (PDK2) and pyruvate dehydrogenase kinase 4 (PDK4), were also significantly downregulated in the HF compared to the normal mice and their levels were further downregulated after exercise. Whereas, there was no significant change observed for the other PDK isoforms. The verification of NAD(+)-consuming enzymes exhibited a dramatic increase of poly [ADP-ribose] polymerase 3 (PARP3) after exercise in HF mice but not poly [ADP-ribose] polymerase 1 (PARP1).

## Discussion

Overall, this study provides a global view of exercise-induced protein-level alterations by LC-MS methods in the murine model of MI-induced HF. The MS analysis finally reveals that the significant shift of 1,304 functional proteins in the adaptive regulation was induced by exercise in a failing heart. The differential proteins were mainly related to cardiac metabolism especially the structure and function of mitochondria. Further confirmation experiments by targeted MS analysis demonstrated the significant downregulation of the component (PDC E1α; PDC E1β) and activity-regulating enzyme (PDK2; PDK4) of PDC and the significant upregulation NAD(+)-consuming enzymes (PARP3). Generally, illustration of the cardiac metabolism-related changes after regular exercise further deepens our understanding of the cardio-regulation mechanisms of aerobic exercise in HF, which may prompt better use of this non-pharmacological intervention and lead to novel alternative therapeutic targets for HF patients.

We employed advanced MS strategies with high sensitivity for this proteomic study. The results were generally reliable based on the repeatability and accurate discrimination between groups. As far as we know, this is the first comprehensive study to explore the effect of exercise on ischemic-induced HF at the molecular level, although the results need to be further verified in humans. Many previous studies have shown the beneficial effect of exercise training on HF, but our understanding of its mechanism is still insufficient due to some discrete individual mechanism studies. Through the analysis of global protein alterations, the study found significant differences in a large number of metabolism-related proteins after 4 weeks of aerobic exercise, especially the proteins involving mitochondrial metabolism, hinting at the crucial role of metabolism remodeling in the regulation of exercise on HF. PDC, which has a central effect on the control of anaerobic metabolism or aerobic metabolic flux, has also been verified to have significant alteration, which suggests that the aerobic metabolism impaired in HF condition after exercise has undergone a significant change after regular exercise.

PDC is the gatekeeper enzyme linking glycolysis and the Krebs cycle by catalyzing the oxidative decarboxylation of pyruvate to acetyl-CoA in mitochondria and comprises three proportional catalytic components including pyruvate dehydrogenase (E1), dihydrolipoamide transacetylase (E2), and dihydrolipoamide dehydrogenase (E3) (24, 25). PDC is subject to inactivation at E1α by four PDK isoforms (PDK 1–4) (24). The E2-lipoyl domains serve as the binding and integrational regions between the regulatory enzymes and PDC (26). A growing body of evidence proved the vital role of the adaptive change in the total level and activity of PDC understanding severely impaired energy metabolic conditions in a failing heart. Recently, an elaborate study comprehensively examined the expression of PDC component proteins and its regulatory proteins found the increased expression levels of E1α, diminished expression of PDK4, and indifferent levels of E1β, PDK1, and PDK2 in the myocardia of HF patients compared to the nonfailing heart, which support sustained adaptive capacity for PDC to facilitate glucose metabolism facing the energy deficiency condition in the failing heart (27). Our results in the mice model are generally similar to this finding in human myocardium except the increased level of E1β, suggesting the consistency of PDC regulation in different species and further serving as a confirmation of our results in the HF condition. More importantly, we further supplemented that exercise could normalize the expression of E1α and E1β to nonfailing condition and further decreased the expression level of PDK2 and PDK4. To date, this is the first study revealing the exercise-induced regulation of PDC activity in the HF condition.

Generally, the complex regulation of PDC includes the long-term transcriptional regulation of component subunits or regulatory enzymes and short-term enzymatic activity regulation by reversible phosphorylation or metabolic intermediates (28). Our study found the transcriptional regulation of both component subunits and PDK after exercise. The expression of four PDK isoenzymes is regulated diversely and the changeable characteristic of PDK2 and PDK4 have been identified previously (29). PDK1 is mostly sensitive to low oxygen supply and the expression of PDK1 is generally activated by hypoxia-inducible factor 1 (HIF-1) in hypoxic conditions acting to the shunting of glucose metabolites away from the mitochondria in the case of ROS accumulation (30, 31). Expression of the other three PDKs are all directly under the upstream regulation by the peroxisome-proliferator-activated receptor (PPAR), a critical factor involved in the control of metabolism (32). While they are sensitive to various stimulating factors, which, respectively, are high concentration of NADH and high acetyl-CoA to CoA ratios for PDK2, high concentration of ATP for PDK3, and energy deprivation for PDK4 (26–28). In our study, the expression of PDK1 was unchanged in the exercise-treated group, suggesting that PDH expression is not significantly modulated by the oxygen supply condition of myocardium. The expression level of PDK3 is too low to be measured due to the overly high affinity to PDC (33). The co-instantaneous downregulation of PDK2 and PDK4 suggests the metabolic markers conventionally believed didn't exert a driving role in the transcriptional regulation of PDC considering the elevated ATP level after exercise in the HF mice (19). By which the expression of PDK2 and PDK4 are regulated and whether PPAR plays dominant roles deserve future works. Subunits of PDC will be downregulated when deprivation or diminishment of energy supplement and vice versa (34). Hence, a higher level of E1αin HF serves as an adaptive response to the energy depletion condition and its normalization after exercise may represent positive feedback after the relative restoration of energy supplement. Broadly speaking, lower expression of PDK suggests a higher proportion of active PDC, whereas the lower E1α and E1β suggest a lower total PDC level. Combined with our previous findings that the glucose metabolism was significantly elevated after exercise (19), we speculated that the total activity of PDC increased after exercise and the rapid control of PDC activity by PDK-mediated in activation rather the PDC component proteins play essential roles in the biological process.

PDC has been increasingly studied as a promising intervention target to restore cardiac function via regulating the oxidation of glucose (35). A recent study on a PDC stimulator, dichloroacetate (DCA), proved its therapeutic effects in restoring the glycolytic flux, maintaining the levels of ATP and improving cardiac function in the chronically hypoxic hearts by inhibiting PDK in mitochondria (36). However, the short half-life limits the clinical practice of DCA in a large scale and diverse drugs targeted on PDC are needed. Exercise has also been shown to regulate metabolism by increasing the expression levels of glycolytic oxidation-related enzymes (37). However, previous evidence on the role of exercise on PDC in HF condition is limited. Our study fills the gaps for the first time and found that regular aerobic exercise regulates PDC not only by the expression of its functional subunits but also the activity-related enzymes. The results enlighten us not only about the molecular insight of exercise on HF but also about future therapeutic selection on PDC. Except the nonpharmacological treatments, more drug targets could be identified by further exploring delicate molecular mechanism for the expression changes and multi-target drugs on PDC may also be a selection.

Nicotinamide adenine dinucleotide (NAD) is a cofactor for energy metabolism in redox reactions. The reduced form (NADH) contributes most electrons to the respiratory chain and the oxidized form (NAD+) is commonly shared as a substrate by several enzymes including the PARPs (38), which play pivotal roles in cardiac metabolism by the function as metabolic sensors (39, 40). PARP, as a ubiquitous nuclear protein, is mainly responsible for DNA repair of injured cells as pathophysiological activation (41) but can also lead to NAD^+^ depletion and cell death (42). Numerous studies have focused on the regulation of NAD homeostasis in cardiac diseases (43). For example, it was found that inhibition of PARP1 exerted cardioprotective roles in post-MI mediated possibly by attenuating cardiac fibrosis, regulating autophagy or reducing apoptosis in some pre-clinical trials (44, 45). In line with previous studies, we observed the decreasing expression level of PARP1 and PARP3 in the MI-induced HF mice model. The upregulation of PARP1 after regular exercise was not verified in subsequent experiments. However, the expression of PARP3 is significantly upregulated in the confirmatory experiment. Considering the detrimental effects of the PARP's over-activation, we can't exclude the possibility that the overexpression of PARP3 after exercise will exacerbate cardiac fibrosis like PARP1 (46). This may be the unusual obstacle hanging over the beneficial role of regular exercise on HF and we should treat it seriously.

## Limitations

Major limitations of our study reflect on the exercise training mode and HF model. More exercise types including resistance training or wheel-running, diverse training duration, and intensity may induce different proteomic alterations in the same HF model. Moreover, repeatability in other nonischemic HF models of the metabolic alterations in the current study remains elusive.

## Conclusion

Our study exhibits the shift of proteomic profiles induced by aerobic exercise in the setting of MI-induced HF, which furnishes not only the systematic insight of the influence of aerobic exercise on HF but also ample molecular targets for future mechanism's experiments. The significant changes of several functional proteins involving energy metabolism support the pivotal role of metabolic remodeling in the regulation of exercise on HF. Adaptive regulations of PDC induced by exercise manifested the downregulating expression of both PDC protein and PDKs. The seemingly reverse regulation suggests the possibly dominant role of PDK in the activation of PDC and further work to elucidate the mechanism of the altered expression response to exercise treatment in the setting of HF is of great benefit for the mitochondrial energy regulation and viable novel drug developments. Further study on the PARP3 is also essential, especially its effect on the cardiac function.

## Data Availability Statement

The raw data supporting the conclusions of this article will be made available by the authors, without undue reservation.

## Ethics Statement

The animal study was reviewed and approved by Zhongshan Hospital, Fudan University, China.

## Author Contributions

SM: conceptualization, project administration, and writing-review and editing. HJia: methodology, project administration, and writing-review and editing. LZ: methodology, project administration, and data analysis. ZX: investigation, data curation, writing-original draft, and writing-review and editing. JZ: data curation and writing-review. AS: investigation, data curation, and writing-review. HJin: resources and data curation. JG: supervision, project administration, and writing-review and editing. All authors contributed to the article and approved the submitted version.

## Funding

This research received a grant from Major Research Plan of the National Natural Science Foundation of China (91639104), a grant to AS from the National Science Fund for Distinguished Young Scholars (81725002), National Natural Science Foundation of China (81800348), and Shanghai Science and Technology Commission(No. 19JC1411302).

## Conflict of Interest

The authors declare that the research was conducted in the absence of any commercial or financial relationships that could be construed as a potential conflict of interest.

## Publisher's Note

All claims expressed in this article are solely those of the authors and do not necessarily represent those of their affiliated organizations, or those of the publisher, the editors and the reviewers. Any product that may be evaluated in this article, or claim that may be made by its manufacturer, is not guaranteed or endorsed by the publisher.

## Abbreviations

HF: heart failure
MI: myocardial infarction
MS: mass spectrometry
LC: liquid chromatography
GO: gene ontology
DEPs: differentially expressed proteins
ATP: adenosine triphosphate
NAD: nicotinamide adenine dinucleotide
PDC: pyruvate dehydrogenase complex
PARP3: poly [ADP-ribose] polymerase 3
DCA: dichloroacetate.
